# Study of Nano-Mechanical Performance of Pretreated Natural Fiber in LDPE Composite for Packaging Applications

**DOI:** 10.3390/ma13214977

**Published:** 2020-11-05

**Authors:** Muhammad Sulaiman, Tanveer Iqbal, Saima Yasin, Hamayoun Mahmood, Ahmad Shakeel

**Affiliations:** 1 Department of Chemical, Polymer & Composite Materials Engineering, University of Engineering and Technology, Lahore (New Campus), Kala Shah Kaku-39020, Pakistan; m.sulaiman@uet.edu.pk (M.S.); tanveer@uet.edu.pk (T.I.); drsaima@uet.edu.pk (S.Y.); engr.hamayoun@uet.edu.pk (H.M.); 2 Department of Hydraulic Engineering, Faculty of Civil Engineering and Geosciences, Delft University of Technology, Stevinweg 1, 2628 CN Delft, The Netherlands

**Keywords:** lignocellulosic biomass, pretreatment, biocomposite, nanoindentation, hardness, modulus

## Abstract

In this work, the effects of chemical pretreatment and different fiber loadings on mechanical properties of the composites at the sub-micron scale were studied through nanoindentation. The composites were prepared by incorporating choline chloride (ChCl) pretreated rice husk waste (RHW) in low-density polyethylene (LDPE) using melt processing, followed by a thermal press technique. Nanoindentation experiments with quasi continuous stiffness mode (QCSM) were performed on the surface of produced composites with varying content of pretreated RHW (i.e., 10, 15, and 20 wt.%). Elastic modulus, hardness, and creep properties of fabricated composites were measured as a function of contact depth. The results confirmed the appreciable changes in hardness, elastic modulus, and creep rate of the composites. Compliance curves indicated that the composite having 20 wt.% of pretreated RHW loading was harder compared to that of the pure LDPE and other composite samples. The values of elastic modulus and hardness of the composite containing 20 wt.% pretreated RHW were increased by 4.1% and 24% as compared to that of the pure LDPE, respectively. The creep rate of 42.65 nm/s and change in depth of 650.42 nm were also noted for the composite with RHW loading of 20 wt.%, which showed the substantial effect of holding time at an applied peak load of 100 mN. We believe that the developed composite could be a promising biodegradable packaging material due to its good tribo-mechanical performance.

## 1. Introduction

In recent years, the focus of researchers has been diverted towards the development of lignocellulosic fiber (LF)-reinforced polymeric composites or biocomposites to replace the non-biodegradable composites because of their eco-friendly nature, low cost, low wear resistance, and good mechanical performance [1,2,3,4]. Nowadays, biocomposites have been widely utilized in various sectors, such as automotive, aerospace, construction, and packaging [5]. It was reported that the biodegradability and renewability of polymeric composites enhance via the addition of LF [6,7]. Utilization of the fiber reduces the competition between chemical and material industries. Consequently, the usage of LF for reinforcement of polymeric matrices is a highly promising and attractive approach for promoting the concept of sustainable development [8].

Various LFs such as rice husk waste, wheat straw, corn cob, bagasse, and cotton stalk have been used to reinforce the polymeric matrix due to improved mechanical properties, nonabrasive nature, easy processing, and low cost as compared to synthetic fibers [7,9]. Basically, LF is composed of three main components, these include cellulose, hemicellulose, and lignin [10]. Lignin has a complex aromatic structure that is connected through a covalent bond with cellulose and hemicellulose, which causes rigidity of the fiber [11]. Lignin in LF is a main cause of poor interfacial adhesion between fiber and polymer matrix [12]. The hydrophilicity of LF is due to hemicellulose and amorphous part of cellulose molecules [13]. LF shows dimensional instability because of its heterogeneous structure and hydrophilic behavior that causes significant machinability problems and weak mechanical properties related to biocomposites [8].

As implicated from the above discussion, LF is not a problem-free alternative. Therefore, pretreatment is the necessary and crucial step for the effective utilization of LF in biocomposite fabrication and other value-added products. Pretreatment, which not only reduces the lignin content but also decreases the hydrophilic nature of the fiber by removing hemicellulose [4,11]. Many pretreatment methods have been exploited for breaking the complex lignocellulosic structure for the removal of lignin, pectin, and waxy impurities [14]. Different pretreatment methods are usually used, such as physical, chemical, and biological methods [15]. Physical techniques involve steam explosion, size reduction, and ultrasonic radiations that reduce the crystallinity of the fiber but do not remove the lignin and demands high energy [4]. Biological methods use micro-organisms and enzymes but are very sensitive and time-consuming processes [4,15]. Chemical pretreatment uses different chemicals, such as acid, alkali, and organic solvents. Pretreatment of the fiber with these chemicals is not so much suggested because of their adverse environmental and health hazard effects [16]. Extreme process conditions such as high temperature and pressure are required for surface modification of the fiber with the above-mentioned methods [14]. Presently, researchers are focusing on environmentally friendly and economical chemicals to pretreat the LF with the goal of lignin and impurities free fiber [17,18]. Choline chloride (ChCl) is emerging as a new solvent for lignocellulose processing that ultimately stimulates the concept of the green environment. ChCl is being used in various applications, including lignocellulosic fiber pretreatment. It is used as a hydrogen bond acceptor for deep eutectic solvents [17]. ChCl has been utilized for lignocellulose fractionation, showing improved cellulose digestibility and efficient removal of lignin [17,18]. Additionally, ChCl can be recycled and is reusable without degradation [18].

In addition to the above-mentioned attributes, interfacial bonding between the polymeric matrix and LF plays a vital role in determining the surface mechanical properties of biocomposites [19]. Surface mechanical properties, such as elastic modulus, hardness, and creep behavior are difficult to measure through conventional characterization techniques due to the anisotropic behavior of LF [3]. Anisotropic behavior is the main reason for the dimensional instability of LF and resultant biocomposites. LF used for biocomposite applications are in the range of micron-meters [6]. Hence, the measurement of mechanical properties of biocomposites is still a challengeable task at the sub-micron scale without the destruction of material.

Nanoindentation is the most powerful and useful technique to analyze the nano-mechanical properties of biocomposites [3]. A nanoindenter provides quantitative data, which is the good source of information related to matrix and reinforcement materials in the biocomposites [20]. This technique has been extensively used for analyzing the mechanical properties of polymers [7]. An indenter in the nanoindenter, controlled by a high-resolution instrument, penetrates the surface of the material with a certain rate and afterward unloaded. Hardness and modulus were calculated using load-displacement data obtained through loading–unloading cycles [21]. In 2007, Lee et al. measured the hardness and modulus of the fiber-reinforced polymeric composite [21]. In 2017, Kavouras et al. explored the effect of microstructure on the fiber-based composite using nanoindentation. The service life of the biocomposite is significantly affected by the creep characteristic of the constituent particles. Tehrani et al. determined the time-dependent behavior of epoxy nanocomposites through nanoindentation [21]. Mechanical properties of polychloroprene composites based on flax fiber were also reported [7].

To the best of our knowledge, limited published work is available describing the effects of pretreated LF on surface mechanical properties of biocomposites. Therefore, the main objective of this study was to investigate the mechanical properties (hardness, elastic modulus, and creep behavior) of biocomposites reinforced with ChCl pretreated rice husk waste (RHW) through nanoindentation. Particularly, the impact of pretreated RHW loading on the mechanical properties of biocomposites was studied. Nano-mechanical properties of biocomposites were also conferred as a function of contact depth using a nanoindenter.

## 2. Experimental Section

### 2.1. Materials

RHW was collected from a local rice mill near Lahore. Choline chloride (ChCl) and low-density polyethylene (LDPE) of 0.93 g/cm^3^ were purchased from Sigma-Aldrich, Germany and Korea Chemicals Ltd., respectively. Distilled water was taken from the chemical process laboratory at the Chemical Engineering Department, UET Lahore (New Campus), Pakistan.

#### 2.1.1. Pretreatment of RHW and Fabrication of Biocomposite

**Figure 1:**
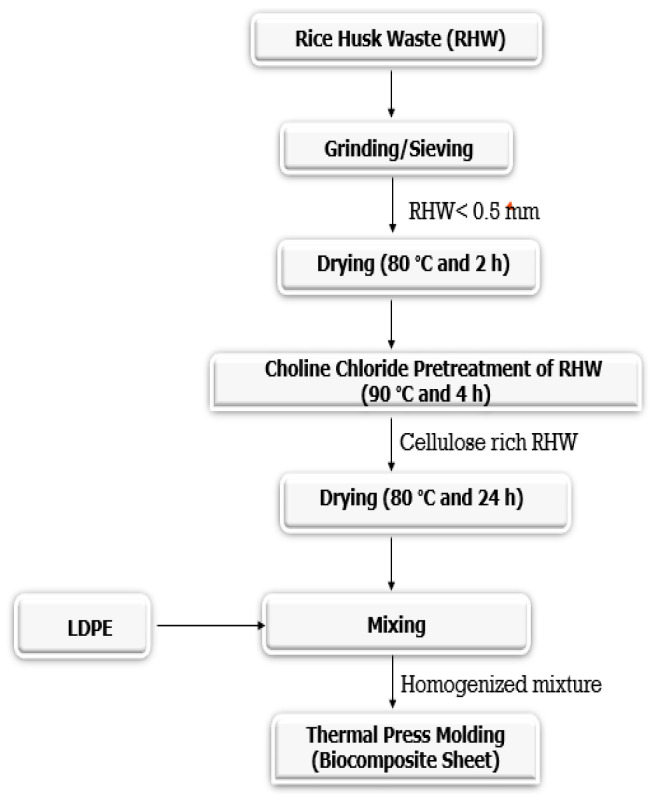
Schematic representation of biocomposite fabrication.

RHW was crushed and sieved to obtain the particle size of ≤ 0.5 mm using a sieve shaker (having U.S. Taylor standard screen, mode: shaking; manufacturer: Filtra Vibracion S.L., Badalona, Spain) and dried in an oven (DAIHAN Labtech Co. Ltd., Namyangju Republic of Korea. LDO-030N, convection type dryer) at 80 °C for 2 h. Dried RHW was pretreated with aqueous choline chloride of 25% (*w*/*w*) in the water bath (model: WTB15, Memmert GmbH + Co., KG, Schwabach, Germany) at 90 °C for 4 h at 150 rpm. Pretreated RHW was properly washed with distilled water (2 to 3 times) and dried in the electric oven at 80 °C for 24 h. For the fabrication of biocomposite samples, pretreated RHW with different biomass loadings (10, 15, and 20 wt.%) were incorporated in LDPE at 115 to 130 °C for 10 min and 300 rpm using an internal mixer (Banbury internal mixer, model: SBI-35L, Well Shyang Machinery Co., Ltd., Taiwan). After homogenizing, a compounded mixture of each sample was thermally pressed (hydraulic platen press, Hartek Technologies Ltd., Guangzhou, China) in a mold of 10 cm × 10 cm × 0.2 cm at 180 °C and 12 MPa for 10 min. Later, the samples were cooled at room temperature to obtain the biocomposite sheet with a target density of 0.4 g/cm^3^. The schematic representation of biocomposite fabrication is depicted in Figure 1. Moreover, RHW loading (wt.%) in the LDPE matrix material to produce the biocomposite samples is shown in Table 1. Furthermore, lignin content was determined for untreated and pretreated RHW, as described by Mahmood et al. [22]. Lignin content was reduced from 19.5 wt.% to 14.3 wt.% after the choline chloride pretreatment.

#### 2.1.2. Characterization

Nanoindentation, also called the depth-sensing method, was proposed by Oliver and his co-workers in 1992 and is used to determine the surface mechanical properties of solid materials [23]. The contact compliance method is basically adopted in the nanoindentation for evaluating the data analysis to avoid the error occurred during the conventional hardness technique [24]. Reaction force on the indenter was measured as a function of imposed depth by applying the contact compliance method, or vice versa, creating the loading–unloading cycles during indentation on the material surface [23]. Consequently, elastic modulus and hardness values are measured using data obtained through loading and unloading indentation cycles [25]. A nanoindenter (Zwick GmbH & Co. KG, Ulm, Germany) was used for performing the normal indentation experiments on the surface of fabricated biocomposite samples. The quasi continuous contact stiffness (dynamic mode) method and Berkovich indenter of three-sided pyramidal geometry with a diamond-shaped tip were utilized to determine the mechanical properties of the samples at the nanoscale. Elastic modulus (stiffness) and hardness values were determined by the fabricated samples without measuring the area of indentation. Basically, indenter tip geometry and depth of penetration is used for measuring the actual indentation area of the sample in the contact compliance mode [25]. An indenter was applied at a maximum force of 100 mN on the surface of each sample to achieve the maximum penetration depth in the tested material. The creep behavior of the produced samples was also investigated via holding the indenter for 20 s at peak load. There were 36 indents selected with a spacing of 60 µm on each sample. Overlapping of residual stress produced around each indent was avoided by keeping the indent spacing constant. A discrete loading and unloading cycle of each indent on the sample was acquired.

## 3. Results and Discussion

### 3.1. Load Displacement Curves

**Figure 2:**
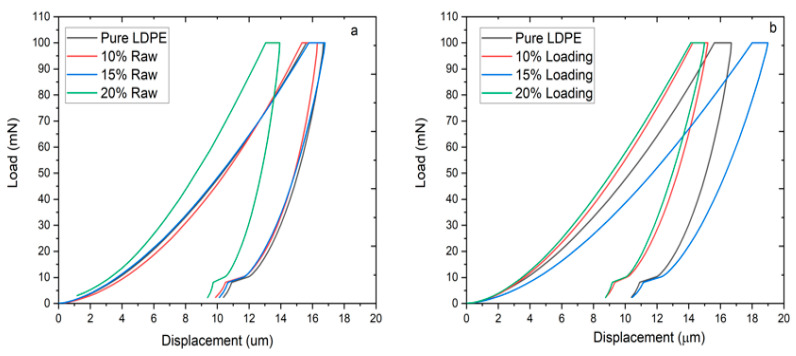
The contact compliance curves of pure LDPE and the biocomposites reinforced with (**a**) raw RHW and (**b**) ChCl pretreated RHW with different loadings (10, 15, and 20 wt.%) at a peak load of 100 mN.

Figure 2a,b depicts the loading–unloading curves of biocomposite sheets produced at a different biomass loading. The loading section of all the samples was started at zero and reached a maximum depth at a peak load of 100 mN. Though, the unloading section was ended between 8 µm to 12 µm after the creep section. However, zero-depth was not achieved due to the plastic nature of the biocomposites. In the case of the pure LDPE sheet, a maximum contact depth of 15.7 µm was achieved, as evidently shown in Figure 2a,b. Whereas contact depth of 14.1, 15.4, and 13.1 µm were obtained after the loading of 10, 15, and 20 wt.% of raw RHW in the LDPE matrix, respectively. The obtained values revealed that the penetration depth in biocomposites was reduced after the addition of raw RHW in LDPE. Though, in the case of ChCl pretreated RHW loading, contact depth of 14.4, 18.0, and 14.7 µm were achieved with the loading of 10, 15, and 20 wt.% of RHW. It is evident from Figure 2a that the biocomposite produced with 15 wt.% of ChCl pretreated RHW is significantly softer than other biocomposites. This behavior may be attributed to the fact that RHW was not uniformly distributed and improperly mixed in LDPE, indicating poor interfacial adhesion between LDPE and pretreated RHW [1]. The contact depth of the biocomposite based on 20 wt % pretreated RHW was increased by 12% as compared to untreated RHW (20 wt.% loading). It may be due to the presence of void spaces or some crack produced during the synthesis of the biocomposite. Whereas, almost the same contact depth in composite was reinforced with 10 wt.% of untreated or pretreated RHW was observed. It could have happened because of the wrapping of a small quantity of RHW in LDPE.

### 3.2. Hardness and Modulus

**Figure 3:**
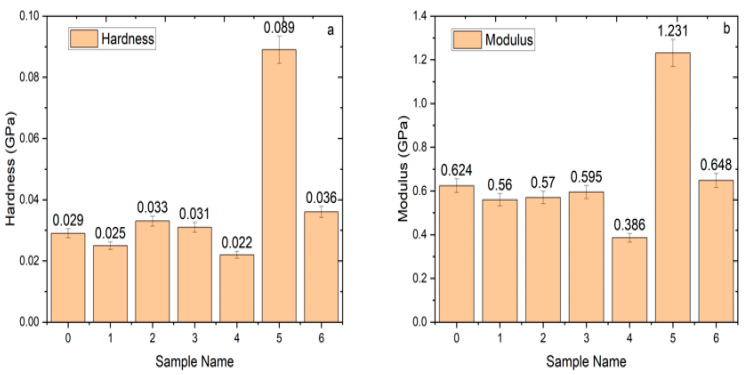
(**a**) Hardness and (**b**) modulus comparison of biocomposite sheets based on untreated and ChCl pretreated RHW.

**Figure 4:**
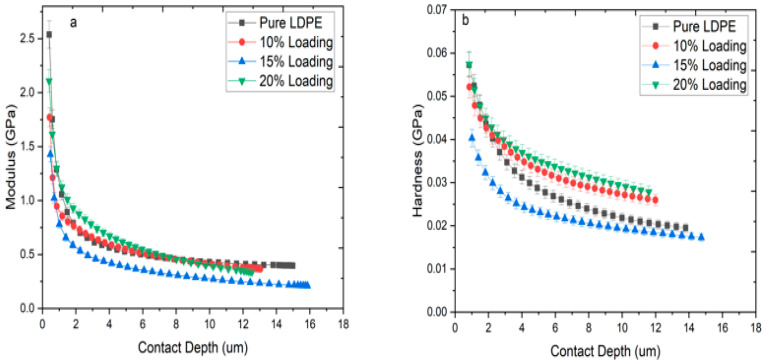
(**a**) Indentation modulus and **(b**) hardness as a function of contact depth for pure LDPE and ChCl pretreated RHW biocomposites with different RHW loadings (10, 15, and 20 wt.%) at 100 mN.

Figure 3a,b shows the indentation hardness (H) and elastic modulus (E_s_) of pure LDPE, raw, and ChCl pretreated RHW reinforced biocomposite sheets with standard deviation. It is clearly indicated in Figure 3a,b that stiffness and hardness of the biocomposites were decreased by the addition of ChCl pretreated RHW compared to raw RHW loading. It might have happened due to the removal of lignin material, which is the main cause of stiffness in the RHW [12]. The hardness and modulus values of the biocomposite produced from 20 wt.% untreated RHW were significantly higher as compared to all other sheets. This response may be linked to the presence of impurities, such as lignin, pectin, and waxy substances in untreated RHW. Moreover, hardness and modulus of the biocomposite based on 20 wt.% RHW loading, either raw or ChCl pretreated, showed the highest values among all the produced sheets. It is evidenced by good interfacial bonding of RHW with LDPE. Figure 4a,b illustrates the H and E_s_ variation that occurred from the top surface to the bulk region of ChCl pretreated RHW reinforced biocomposites. It was observed that both H and E_s_ were significantly decreased up to 6 µm of contact depth, which represented the top surface of the sheets. It could be concluded that the top surface of the sheets was rough and uneven, due to which a large variation in hardness and elastic modulus was observed. This variation might have happened because of surface changes due to environmental effects, poor determination of the top surface, and a defect in indenter tip geometry [26]. However, no abrupt changes in H and E_s_ of the biocomposites reinforced with pretreated RHW were noted from 6 to 16 µm of contact depth in Figure 4a,b, indicating the amorphous soft region of the sheets [27]. Indentation modulus of 0.624 GPa was obtained for the LDPE sheet, as shown in Figure 4a, representing the compact and semi-crystalline structure of LDPE. The lowest values of modulus and hardness of the biocomposite reinforced with 15 wt.% of ChCl pretreated RHW were noted among all produced sheets. It may be due to the greater possibility of improper distribution of RH, ultimately leading to the formation of agglomerates [19]. Generally, high elastic modulus corresponds to high hardness value and vice versa [3]. An interesting fact was noted—the sheet with 10 wt.% of ChCl pretreated RHW loading showed higher hardness and lower elastic modulus as compared to the LDPE sheet in Figure 4a,b. It might be due to the small quantity of RHW that was wrapped in the LDPE matrix.

### 3.3. Hardness to Modulus Ratio (H/E_s_)

**Figure 5:**
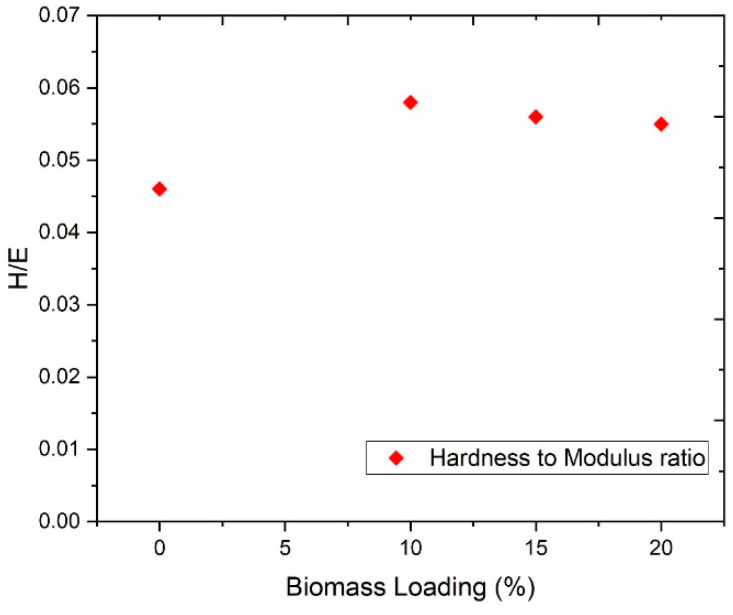
Hardness to modulus index of biocomposites with different pretreated RHW loadings (10, 15, and 20 wt.%).

Wear resistance of material is usually evaluated through H/E_s_ [26]. Basically, this ratio defines the relative plastic/elastic behavior of the material under applied load and deformation [28]. It could be used for measuring the fracture toughness, elastic strain to failure, and critical yield stress for plastic deformation. Figure 5 depicts the H/E_s_ ratio of the biocomposites reinforced with ChCl pretreated RHW of different loadings. The biocomposites showed the highest value of this ratio as compared to the LDPE sheet, which indicated good wear resistance. A higher value of this ratio means less contribution of E compared to H. In 2019, Payman Nikaeen reported that the H/E_s_ ratio of nanofiber reinforced low-density polyethylene through nanoindentation [26]. However, this H/E_s_ ratio was observed to decrease with the increase of pretreated RHW loadings from 10 to 20 wt %, showing the gradual enhancement of stiffness as biomass content increased.

### 3.4. Creep Behavior

**Figure 6:**
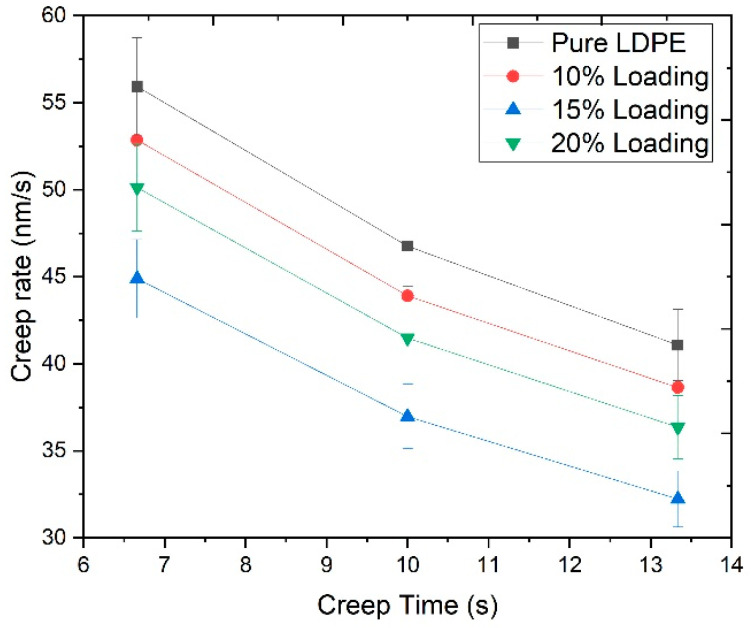
Creep rate vs. holding time of pure LDPE and the biocomposites reinforced with ChCl pretreated RHW loadings (10, 15, and 20 wt.%) at a peak load of 100 mN.

The biocomposites showed viscoelastic behavior due to the semi-crystalline and amorphous structure of its constituent particles. Stress, holding time, and temperature are the most important factors that significantly affect the viscoelastic behavior of the composite [29]. Figure 6 presents the creep rate of pure LDPE and pretreated RHW reinforced biocomposites. The creep rate showed a decreasing trend at a constant peak load of 100 mN. The creep rate of the pure LDPE decreased from 55.9 to 40.9 nm/s, whereas the creep rate of the biocomposites was lower than the pure LDPE sheet because the pretreated RHW contained higher contents of cellulose. This cellulose had a more compact and crystalline structure; therefore, minimum variation in depth was observed over constant load. The creep resistance of the biocomposites was increased via the addition of ChCl pretreated RHW in the LDPE. Reduction in the creep rate (%) was noted as 5.9%, 11.3%, and 22.1% with 10, 20, and 15 wt.% of pretreated RHW, respectively. It was clearly indicated from the reduction in the creep rate that the biocomposite with 15 wt.% pretreated RHW loading exhibited a lower creep rate as compared to the other samples. It might have happened because of the non-uniform and improper mixing of RHW in LDPE, leading to the formation of agglomerates. Consequently, interfacial adhesion was not only interrupted, but slippage of RHW particles occurred that ultimately affected the creep resistance of the material [30].

### 3.5. Effect of Holding Time on Depth

**Figure 7:**
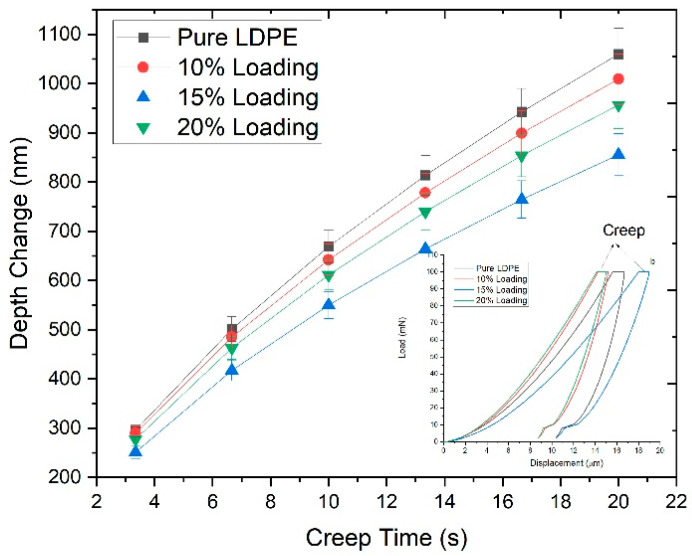
Depth change vs. holding time of pure LDPE sheet and the biocomposites reinforced with ChCl pretreated RHW loadings (10, 15, and 20 wt.%) at peak load of 100 mN for 20 s.

Figure 7 represents the creep time effect on the depth change in biocomposites having ChCl pretreated RHW at a peak load of 100 mN for 20 s. The creep depth was decreased to a significant level during creep time at peak load by applying the Oliver–Pharr method. The creep depth of the pure LDPE sheet varied largely from 291.3 to 1052.2 nm during holding time due to its semi-crystalline structure. It is shown in Figure 7 that change in creep depth also happened in biocomposites but less than the pure LDPE because RHW induced the stiffness in the biocomposites [31]. Furthermore, it could be predicted that the elastic behavior of RHW was reduced due to partial removal of amorphous polymers (hemicellulose and lignin) by ChCl pretreatment [32]. Therefore, maximum creep depths of 1004.1 nm, 851.6 nm, and 956.5 nm were noted in the biocomposites having 10, 15, and 20 wt.% of ChCl pretreated RHW loadings, respectively.

## 4. Conclusions

The impact of choline chloride (ChCl) assisted processing of rice husk waste (RHW) on surface mechanical properties of produced biocomposites at nanoscale was reported. The hardness, elastic modulus, and creep rate of the composites were appreciably changed as a function of operational parameters such as peak load, contact depth, and holding time. The ChCl pretreatment showed a profound impact on the contact stiffness and indentation hardness of RHW based composites under different fiber loadings. The maximum values of elastic modulus and indentation hardness were exhibited for 20 wt.% of RHW loading. Interestingly, the same fiber loading provoked the lowest indentation depth under peak load. The creep rate and change in depth of the composites considerably varied with holding time, which may have revealed the uniform mixing of fiber in the matrix phase. The obtained H/E_s_ ratio indicated that the plastic behavior of the composites was enhanced with fiber loading.

## Figures and Tables

**Table 1:** Raw and ChCl pretreated RHW loading (wt.%) in the low-density polyethylene (LDPE) matrix material, along with their sample ID.

Sample Name	Matrix Material	Rice Husk Waste (RHW) Loading
0	LDPE	0 wt.% RHW
1	LDPE	10 wt.% raw RHW
2	LDPE	10 wt.% ChCl pretreated RHW
3	LDPE	15 wt.% raw RHW
4	LDPE	15 wt.% ChCl pretreated RHW
5	LDPE	20 wt.% raw RHW
6	LDPE	20 wt.% ChCl pretreated RHW
